# Impact of China’s environmental decentralization on carbon emissions from energy consumption: an empirical study based on the dynamic spatial econometric model

**DOI:** 10.1007/s11356-022-18806-x

**Published:** 2022-03-30

**Authors:** Xianzhao Liu, Xu Yang

**Affiliations:** School of Resource, Environment and Safety Engineering, Hunan University of Science and Technology, Xiangtan, 411201 Hunan China

**Keywords:** Environmental decentralization, Carbon emission, Spatial dependence perspective, Dynamic spatial econometric model

## Abstract

Facing the growing problem of carbon emission pollution, the scientific and reasonable division of environmental management power between governments is the premise and institutional foundation for realizing China’s carbon emission reduction target in 2030. In this article, we directly assess the degree of environmental decentralization according to the allocation of environmental managers among different levels of government. By incorporating fiscal decentralization indicators, the provincial panel data and dynamic spatial econometric model are used to empirically test the impact of environmental decentralization on carbon emissions from a spatial perspective. The results show that (1) China’s provincial carbon emissions have significant inertia dependence and spatial path dependence. The increase (decrease) of provincial carbon emissions will lead to the increase (decrease) of carbon emissions in neighboring regions. (2) At the national level, environmental decentralization, environmental administrative decentralization, and environmental monitoring decentralization significantly reduce China’s carbon emissions, while environmental supervision decentralization and fiscal decentralization significantly increase carbon emissions. Similarly, the interaction of environmental decentralization and its decomposition indicators and fiscal decentralization also significantly promotes carbon emissions, and the impact is related to the types of environmental management decentralization. (3) The carbon emission effects of environmental decentralization in different regions are heterogeneous. The inhibition effect of environmental decentralization, environmental administrative decentralization, and environmental monitoring decentralization on carbon emissions in the western region is significantly greater than that in the eastern and central regions, but the inhibitory effect of the interaction of environmental decentralization and its decomposition index and fiscal decentralization on carbon emissions in the eastern region was significantly stronger than that in the central and western regions. The above results provide theoretical support for China to construct a differentiated carbon emission environmental management system from two aspects of regional differences and environmental management power categories.

## Introduction

With the rapid development of social economy and the increasing energy consumption, the world environmental pollution problems have become increasingly prominent. Especially as the important part of environmental pollution, CO_2_ emission (referred to as carbon emission) leads to global warming and ecological environment deterioration, which has become an important factor plaguing socioeconomic development. Under such circumstances, there is no doubt that reducing greenhouse gas emissions and developing low-carbon economy have become important measures for governments to deal with climate change and solve the environmental pollution problem (Halkos and Tzeremes 2013). According to BP World Energy Statistics, China’s carbon emissions have been growing rapidly since 2000 and reached 9.258 billion tons in 2017, accounting for 27.3% of the world’s total carbon emissions (Liu et al. 2020). To improve environmental quality, Chinese government has made a great deal of effective efforts to fulfill national commitments on carbon emission reduction (that is, the carbon emissions per unit of GDP in 2030 will be reduced by 60–65% compared to 2005, and the proportion of non-fossil energy in total primary energy consumption will reach to 20%). Especially in the “13th Five-Year Plan,” China has clearly taken the reform of environmental governance system, the implementation of local government environmental protection responsibility, and the construction of a modern environmental management system as a basic task. At the same time, several reform initiatives also have been proposed, including the establishment of clear responsibilities and powers, environmental protection supervision, and for environmental damage accountability. With the distribution of environmental management power among multi-level governments, increasing attention has begun to be paid to the role of local governments in implementing environmental policies and controlling environmental pollution (Zhang et al. 2017a, b; Sigman 2014; Luo and Ling 2020). As an institutional factor, environmental management directly affects environmental quality, so it has always been a research hotspot. At present, most scholars mainly study the factors affecting carbon emissions from the aspects of economic growth, population size, energy consumption, industrial structure, and technology level, while less research has been conducted specifically on the institutional factors affecting carbon emissions (Poon et al. 2006; Al-mulali et al. 2013). However, it is undeniable that a region’s carbon emissions cannot be independent of the environmental institutional factors. Environmental decentralization, as an important part of the environmental management system, will inevitably have a significant impact on regional carbon emissions to some extent.

As for the environmental management system for carbon emission, it can be traced back to the classic environmental federalism theory (a branch of fiscal federalism) in the 1970s (Oates and Schwab 1988). Its meaning can be understood as the de facto decentralization of environmental management, that is, the central government delegates the power of environmental management to local governments, so that local governments have a certain autonomy and decision-making power in environmental management affairs. The core of this theory is how to optimize the allocation of environmental management power among different levels of governments (Cole et al. 2013). Currently, the debate over the impact of environmental decentralization on environmental quality (carbon emissions) is dominated by three different perspectives, namely inhibition, promotion, and uncertainty theory. Some scholars represented by Oyono (2005) and Gray and Shadbegian (2004) argue that environmental decentralization management is not conducive to environmental protection, but rather tends to exacerbate environmental pollution in local and neighboring areas, thereby curbing the improvement of environmental quality. For example, Ran et al. (2020) confirmed that environmental decentralization exacerbated environmental pollution using panel data from 30 provinces in China. Scholars who hold this view mostly expound the harm of decentralization to the environment from the perspective of jurisdictional competition. They believed that the decentralization system distorted the supply mode of environmental public goods, and caused incentive distortions and insufficient constraints (He 2015; Tian and Wang 2018; Wang and Zhang 2014; Ben and Li 2017; Huang 2017). In other words, local officials, in order to obtain sufficient economic benefits and job promotion, often choose to relax environmental regulations for “race to the bottom,” and even misappropriate environmental protection expenditure or form collusion between government and enterprises, leading to inefficient environmental policies and thus aggravating environmental pollution (Kunce and Shogren 2007; Dijkstra and Fredriksson 2010). On the contrary, centralized environmental management can enable central government to provide better environmental public services, thereby reducing supply costs and avoiding the “free-riding” of local governments caused by decentralization (Long and Hu 2014; Liu et al. 2015). However, supporters of decentralization argue that environmental decentralization is more conducive to local governments to provide better environmental services, so as to control environmental pollution and significantly reduce carbon emissions (Fslleth and Hovik 2009; Tan and Zhang 2015; Zou et al. 2019). The reasons for this are as follows. Firstly, the local governments have more information advantages than the central government in the provision of public goods, so as to provide better environmental governance services to residents with high efficiency and low-cost manner (Zou et al. 2019; Lu and Yang 2019; Banzhaf and Chupp 2012). Secondly, the environmental decentralization makes the responsibility and authority of local governments in environmental management affairs more clear, which is more conducive to mobilizing the enthusiasm of residents to exercise environmental supervision power, thereby promoting the transformation and upgrading of industrial structure, and then improving the environmental quality (Li 2018; Goel et al. 2017). Scholars of uncertainty theory believe that under the background of jurisdictional competitive for resource mobility, local governments seek to maximize self-interest rather than social welfare (Besley and Coate 2003). Therefore, environmental decentralization may not only cause “race to the bottom” and have a negative impact on the environment, but also form “race to the top” and have a positive impact. Its impact direction depends on the degree of cross-border pollution of public goods and the heterogeneity of local governments’ preferences (Ran et al. 2020; Ferrara et al. 2014). Besides that, Fredriksson and Wollscheid (2014) found that different forms of environmental decentralization have different impacts. Environmental administrative decentralization (EAD) usually has a positive drive on the environment pollution, while environmental monitoring decentralization (EMD) has a negative effect on the environment. Some scholars also have confirmed that there is an inverted U-shaped relationship between decentralization and environmental pollution (Jacobsen et al. 2012; Li and Liu 2016).

In recent years, with the increasingly prominent environmental problems in China, more and more scholars have begun to pay attention to the impact of Chinese decentralization on carbon emissions. Most of the current findings are consistent with the first view above, which holds that decentralization will increase carbon emissions of the region and the surrounding areas (Lu and Zhang 2016; Zhang et al. 2017a, b). However, under the complex background of “block competition” produced by Chinese-style decentralization reform and “strip competition” produced by political centralization, does the current environmental decentralization promote or inhibit China’s carbon emissions? No definite answer was given (Zhang et al. 2011; Ran et al. 2020).

In summary, there are divergent conclusions about the impacts of environmental decentralization on environmental pollution or carbon emission. We believe that the divergences can be attributed to four main reasons. First, in terms of the description of environmental decentralization indicators, the existing studies mainly use fiscal decentralization indicators to approximately replace environmental decentralization, or indirectly characterize environmental decentralization based on legal system and factual characteristics (He 2015; Huang 2017), ignoring the essential difference between environmental decentralization and financial decentralization (Jacobsen et al. 2012; Deng et al. 2012). In fact, environmental decentralization is an environmental management institution established by the central government through the delegation of environmental protection functions to local governments, reflecting the division of environmental powers with basic environmental public services at the core, while fiscal decentralization emphasizes the division of political centralization and economic decentralization between central and local governments, which hardly reflects the division of responsibility for environmental protection between the central government and local governments. Therefore, the relative independence and uniqueness of environmental protection function determine that fiscal decentralization cannot replace environmental decentralization. If fiscal decentralization is used to approximate the environmental decentralization between governments, it may lead to deviations in the measurement of environmental decentralization, thus affecting the investigation of the real relationship between environmental decentralization and environmental pollution. Second, although some studies have explored the carbon emission effect of environmental decentralization from a spatial perspective and found that environmental decentralization can significantly increase carbon emissions indirectly through job promotion and economic incentives (Bai and Nie 2017; Ran et al. 2020), most scholars ignored the spatial spillover effect of environmental pollution (e.g., carbon emissions), and this effect and spatial correlations have been confirmed by many scholars in the federal and developing countries (Anselin 2001; Cole et al. 2013; Maddison 2006; Poon et al. 2006; Hossein and Kaneko 2013; Liu et al. 2015; Tian and Wang 2018). Thus, in the theoretical and empirical research on the impact of environmental decentralization on regional carbon emissions, if the objective spatial association of carbon emissions is ignored, the research results will be biased. Third, previous studies have not considered the interaction between environmental and fiscal decentralization when analyzing the effects of environmental decentralization on environmental pollution, which makes it difficult for the environmental federalism theory rooted in fiscal decentralization to explain the internal mechanism of pollutant change from the perspective of environmental decentralization. Actually, under the background of fiscal decentralization reform and environmental protection “fragmentation,” fiscal decentralization not only gives local governments’ greater economic autonomy, but also affects the implementation of local environmental protection management powers to a certain extent. Therefore, in order to more accurately investigate the impact of environmental decentralization on carbon emissions, it is necessary to pay attention to the interaction between environmental decentralization and fiscal decentralization on carbon emission under the circumstance of Chinese-style decentralization. Fourthly, the existing studies neglected the spatial heterogeneity of the impacts of environmental decentralization on carbon emissions in different regions of China. That’s because different regions may have different levels of economic development, energy consumption structure, and environmental policies. In addition, the existing panel models take less account of the dynamics and continuity of the explained variables, which may affect the consistent estimation of the effect of environmental decentralization on carbon emission. As a result, there is considerable room for improvement in both logic and accuracy of existing research.

Compared with previous literature, this study may have three contributions. First, based on the methods of Ran et al. (2020), we construct the index that fits China’s environmental decentralization and analyze the impacts of environmental decentralization and its interaction with fiscal decentralization on carbon emission, which expands the application of environmental federalism theory in China’s carbon emissions research. Second, considering the dynamics and continuity of the explained variable (carbon emission), three types of models, such as static panel model, static spatial panel model, and dynamic spatial panel model, are established to test whether the current environmental decentralization really intensifies China’s carbon emissions. Third, considering the heterogeneity of regional economic and social developments, energy consumption structure, and resource endowments as well as the different environmental policies, the regional differences in the impacts (including the direction and degree of impact) of environmental decentralization on carbon emissions in eastern, central, and western China are compared under the background of fiscal decentralization, which will provide a policy reference for promoting the realization of China’s carbon emission reduction target in 2030 and formulating differentiated environmental management strategy.

## Research design

### Model building

In order to empirically examine the carbon emission effect of Chinese-style environmental federalism, referring to the relationship between decentralization and environmental quality proposed by Elhorst (2012), Sigman (2014), and Ran et al. (2020), we constructed a static panel data model, static spatial panel data model, and dynamic spatial panel data model, in which environmental management decentralization and fiscal decentralization were incorporated. The specific settings of the three types of models are as follows:

1 $$\mathrm{ln}{PCO}_{2it}={\beta }_{0}+{\beta }_{1}{CEV}_{it}+\theta {X}_{it}{+\varepsilon }_{it}$$

2 $$\mathrm{ln}{PCO}_{2it}={\beta }_{0}+\rho W\mathrm{ln}P{CO}_{2it}+{\beta }_{1}{CEV}_{it}+\theta {X}_{it}{+\delta }_{i}+{\mu }_{t}+{\varepsilon }_{it}$$

3 $$\mathrm{ln}{PCO}_{2it}={\beta }_{0}+\tau L.\mathrm{ln}P{CO}_{2it}+\rho W\mathrm{ln}P{CO}_{2it}+{\beta }_{1}{CEV}_{it}+\theta {X}_{it}{+\delta }_{i}+{\mu }_{t}+{\varepsilon }_{it}$$

In the formula, *i* and *t* denote provinces ($i=\mathrm{1,2},\cdots ,30$) and years, respectively; ${lnPCO}_{2it}$ is the explained variable, expressed as the logarithm of the provincial per capita carbon emissions; *CEV* represents the core explanatory variables, including environmental decentralization and fiscal decentralization; *X* indicates other control variables affecting carbon emissions; *ε* is the random error term; *δ* and *μ* are individual fixed effect and time fixed effect respectively; *β*_0_, *β*_1_, and *θ* are the estimated parameters of the models. This study mainly focuses on the change of parameter *β*_1_, which describes the direction and degree of the impact of environmental decentralization on carbon emissions.

Among the above three types of models, Eq. (1) is a static panel model, which is mainly used to provide reference for the other two types of models. Equation (2) is a static spatial panel model; that is, considering the spillover effect and spatial dependence (spatial correlation) of carbon emission (Liu et al. 2015, 2018), the spatial lag term of carbon emission $(\rho WlnP{CO}_{2it})$ is introduced on the basis of Eq. (1). Among them, *ρ* is the spatial lag coefficient, which reflects the impact of carbon emissions in surrounding areas on local carbon emissions; *W* is a geospatial adjacency weights matrix (In the research process, three weight matrices are used: geographic adjacency matrix, geographic distance matrix, and economic distance matrix. In comparison, the geographic adjacency matrix is relatively simple and more in line with the characteristics of pollutant spatial spillover. Thus, the geographic adjacency matrix is finally selected as the weight matrix), the value of which is determined according to the Queen’s principle of geographic adjacency. When two regions are adjacent and share common border and vertex, the weight is set as 1; otherwise, it is 0. Further, considering the potential endogeneity of the model itself and the dynamics and continuity of dependent variables, we added the time lag term of carbon emission ($\tau L.lnP{CO}_{2it}$) on the basis of Eq. (2) (τ is the time lag coefficient, indicating the impact of carbon emissions in the previous period on carbon emissions in the current period) and set up a dynamic spatial panel model, namely Eq. (3). Simultaneously, the unconditional maximum likelihood estimation (Elhorst 2012) and first-order difference methods were used to eliminate the fixed effect. In addition, to test the interaction effect of environmental and fiscal decentralization on carbon emissions, an interaction term (${IT}_{it}$) of them was included in Eq. (3), which was extended to Eq. (4), that is:

4 $$\mathrm{ln}{PCO}_{2it}={\beta }_{0}+\tau L.\mathrm{ln}P{CO}_{2it}+\rho W\mathrm{ln}P{CO}_{2it}+{\beta }_{1}{CEV}_{it}+\theta {X}_{it}{+{\beta }_{2}{IT}_{it}+\delta }_{i}+{\mu }_{t}+{\varepsilon }_{it}$$

### Variable measurement

Based on the data of 30 provinces in Chinese mainland (excluding Hong Kong, Macau, Taiwan, and Tibet) in 2003–2017 and referred to the research results on carbon emissions both at home and abroad, the eight major factors affecting carbon emissions were selected as the independent variables to conduct empirical analysis. The definition and measurement of each variable are as follows.

#### Explained variable

For the explained variable, this study used the reference method provided by the IPCC to estimate the per capita carbon emissions in each province based on the consumption of eight major fossil energy sources (raw coal, coke, crude oil, gasoline, kerosene, diesel oil, fuel oil, and natural gas). The specific formula is:

5 $${PCO}_{2it}=\left(\sum\nolimits_{j=1}^{8}{E}_{j}\times {SCC}_{j}\times {CEC}_{j}\times 44/12\right)/{POP}_{it}$$

where *i* and *t*, respectively, stand for province and year, *PCO*_2_ is provincial carbon emissions per capita, *j* is energy type, *E* denotes the consumption of fossil energy, *SCC* is the conversion coefficient of standard coal for fossil energy (Table 1), *CEC* is the carbon emission coefficient of fossil energy (Table 1), 44/12 is the ratio of molecular weight of CO_2_ to molecular weight of carbon, and *POP* is the year-end population of each province.

**Table 1:** The conversion coefficient of standard coal and carbon emission coefficient for eight fossil energy sources

Coefficient	Raw coal	Coke	Crude oil	Gasoline	Kerosene	Diesel oil	Fuel oil	Natural gas
SCC(kg tce/kg)	0.7143	0.9714	1.4286	1.4714	1.4714	1.4571	1.4286	1.3300^*^
CEC(kg/kg tce)	0.7559	0.8550	0.5857	0.5538	0.5714	0.5921	0.6185	0.4483

The unit of conversion coefficient of natural gas is kg standard coal∙m^−3^

#### Core explanatory variables

As one of the core explanatory variables, environmental decentralization means that the central government delegates the power of environmental management to local governments, and endows local governments with certain autonomy and decision-making power in environmental governance, so as to realize the incentive compatibility of central and local governments’ environmental management and the effective supply of public services for environmental protection. Unlike the western environmental federalism system, Chinese-style environmental decentralization has integrity and greater freedom for local environmental management authorization. In addition, China’s environmental protection power is divided in detail, including environmental policy-making, environmental monitoring, and environmental supervision as well as investment in environmental facilities and environmental information services (Qi et al. 2014). Because it is very difficult to construct an environmental decentralization index that comprehensively measures the self-consistency of practice and theory, most of the previous studies indirectly measure environmental decentralization by using indicators such as virtual variables, local independent legal proportion, and fiscal decentralization (Sigman 2014). However, the particularity of environmental management power determines that the above indicators cannot replace environmental decentralization. Only by directly constructing indicators to measure environmental decentralization based on the internal logic of environmental powers can we objectively and accurately reflect the essential connotation of environmental decentralization in China. In this study, the personnel distribution of environmental protection agencies at different levels is used to describe the overall environment decentralization (ED), which is subdivided into environmental administrative decentralization (EAD), environmental monitoring decentralization (EMD), and environmental supervision decentralization (ESD). In addition to considering the availability of data, the use of personnel distribution to characterize environmental decentralization is mainly based on the following reasons: (1) as the carrier of exercising environmental protection power, the personnel of environmental protection institutions can reflect the specific division of environmental power among governments at different levels to a certain extent; (2) the change in the distribution of personnel in environmental protection agencies can reflect the variation in the environmental management system with the division of environmental responsibility as the core; and (3) the essence of environmental decentralization is management decentralization, and personnel distribution can better reflect the essence of environmental decentralization. Therefore, it is scientific and applicable to use the ratio between the number of local and national personnel per capita in environmental protection agencies to characterize the level of environmental decentralization. The calculation formulas of the above decentralization indicators are as follows.

6 $${ED}_{it}=\left[\frac{{SYS}_{it}/{POP}_{it}}{{SYS}_{t}/{POP}_{t}}\right]\times \left[1-\frac{{GDP}_{it}}{{GDP}_{t}}\right]$$

7 $${EAD}_{it}=\left[\frac{{SYSA}_{it}/{POP}_{it}}{{SYSA}_{t}/{POP}_{t}}\right]\times \left[1-\frac{{GDP}_{it}}{{GDP}_{t}}\right]$$

8 $${EMD}_{it}=\left[\frac{{SYSM}_{it}/{POP}_{it}}{{SYSM}_{t}/{POP}_{t}}\right]\times \left[1-\frac{{GDP}_{it}}{{GDP}_{t}}\right]$$

9 $${ESD}_{it}=\left[\frac{{SYSS}_{it}/{POP}_{it}}{{SYSS}_{t}/{POP}_{t}}\right]\times \left[1-\frac{{GDP}_{it}}{{GDP}_{t}}\right]$$

In the formula, subscripts *i* and *t* denote province and year, respectively; ${SYS}_{it}$, ${SYSA}_{it}$, ${SYSM}_{it}$, and ${SYSS}_{it}$, respectively, represent the total number of personnel in environmental protection system, the number of environmental protection administrative personnel, the number of environmental protection monitoring personnel, and the number of environmental protection supervising personnel at provincial level; ${SYS}_{t}$, ${SYSA}_{t},$ ${SYSM}_{t}$, and ${SYSS}_{t}$ are, respectively, the number of personnel in the environmental protection system, the number of environmental protection administrative personnel, the number of environmental protection monitoring personnel, and the number of environmental protection supervising personnel at the national level. ${POP}_{it}$ and ${POP}_{t}$ indicate the year-end population of each province and the whole country, respectively;${GDP}_{it}$ and ${GDP}_{t}$ are the gross domestic product of each province and the whole country, respectively; $\left[1-\left({GDP}_{it}/{GDP}_{t}\right)\right]$ is an economic reduction factor, which is used to deflate the impact of economic scale on the actual degree of environmental decentralization. The greater the above value of ${ED}_{it}$, the higher the degree of environmental decentralization, and other decentralization values (e.g., ${EAD}_{it}$, ${ESD}_{it}$, and ${EMD}_{it}$) also have a similar relationship.

Fiscal decentralization (FD) is the second core explanatory variable. There are two main reasons for taking it as the core explanatory variable. Firstly, the theory of environmental decentralization is rooted in the theory of fiscal federalism. That is, FD is the basis of ED, which affects the implementation of local environmental protection management power to a great extent. Secondly, because previous studies mostly used FD indicator to describe ED, the empirical part of this paper also takes FD as the core explanatory variable to test whether China’s ED and FD are consistent in affecting carbon emissions. Based on the availability of data and referring to the method of Zou et al. (2019), the degree of fiscal autonomy is used to characterize the FD of each province. The formula is ${FD}_{it}={FE}_{it}/{FI}_{it}$, where ${FE}_{it}$ and ${FI}_{it}$ are the fiscal expenditure and fiscal revenue in the budget of each province.

#### Control variables

In addition to institutional factors (e.g., environmental decentralization and fiscal decentralization) affecting carbon emissions, there are many socioeconomic factors influencing carbon emissions. Considering the robustness of the model and in order to control the impact of socioeconomic factors on carbon emissions (Cole et al. 2013; Qi et al. 2014), this study selected the economic development level, population density (*PD*), R&D intensity (*RD*), foreign direct investment (*FDI*), industrial structure (*INDUS*), and trade openness (*OPEN*) as the control variables. Specifically, the economic development level is measured by the logarithm of the per capita GDP (*lnPGDP*) in each province, and the GDP deflator is used to eliminate the impact of price fluctuations. At the same time, the square term of *lnPGDP* is introduced to investigate whether the Kuznets curve of carbon emission exists. The *PD* is expressed by the logarithm of the ratio of the year-end population to the area of corresponding province. *RD* is measured by the proportion of R&D expenditure in GDP of each province. *FDI* is expressed as the proportion of the actually utilized foreign direct investment (converted by the average exchange rate of RMB against the US dollar) in GDP of each province. *INDUS* is measured using the value-added of the secondary industry as a share of GDP. *OPEN* is expressed by the proportion of the total import and export trade of each province in GDP.

### Data sources and variable descriptive statistics

The raw data used to estimate the above indicators were from China Statistical Yearbook, China energy statistical yearbook, China Environmental Yearbook and China Financial Yearbook from 2004 to 2018 (https://navi.cnki.net/knavi/yearbooks/index). As China Environmental Yearbook has not provided the number of provincial environmental protection administrators (but has always provided the data of provincial environmental monitoring personnel and environmental protection supervisors) since 2016, the number of provincial environmental protection administrators in 2016–2017 is obtained through generative adversarial networks (GAN) interpolation (Zhang et al. 2021), and then the number of environmental protection system personnel in each province is obtained by summing environmental protection administrators, environmental protection monitoring personnel, and environmental protection supervisors. Data such as the output value of the secondary industry, the total import and export volume, and FDI, were derived from the statistical yearbooks of all provinces over the years. Among them, the FDI data of Hebei and Gansu provinces came from the Hebei Economic Yearbook and the Gansu Jiangsu Development Yearbook respectively (https://navi.cnki.net/knavi/yearbooks/index). All indicators expressed in monetary units were deflated using the 2000 price index as the base period (FDI data were first converted using the average exchange rate of the RMB against the USD for each year and then deflated using the 2000 price index). The descriptive statistical results of each variable are shown in Table 2.

**Table 2:** Descriptive statistics of all the variables

Variable	Mean	Std. D	Max	Min	Obs
_*lnPCO2*_	1.711	0.551	3.516	0.269	450
*L.lnPCO*_*2*_	1.661	0.546	3.289	0.138	450
*ED*	1.008	0.365	2.347	0.059	450
*EAD*	1.027	0.582	10.612	0.186	450
*EMD*	1.033	0.725	14.203	0.069	450
*ESD*	0.972	0.545	3.503	0.185	450
*FD*	2.248	0.980	7.426	0.197	450
*lnPGDP*	3.031	0.650	4.586	1.246	450
(*lnPGDP*)^2^	9.607	3.9705	21.031	1.551	450
*lnPD*	5.429	1.266	8.249	2.036	450
*RD*	1.871	1.535	9.844	0.172	450
*FDI*	3.197	2.376	10.941	0.054	450
*lnINDUS*	3.812	0.207	2.944	4.202	450
*OPEN*	0.394	0.424	1.891	0.018	450

## Results

### Spatial correlation test for carbon emissions

Testing the existence of spatial correlation of variable is the premise of using dynamic spatial panel model to analyze the impact of environmental decentralization on carbon emissions. At present, most scholars adopt Moran’s *I* index to characterize the spatial autocorrelation of regional variables (that is, a geographical phenomenon in a spatial unit is related to that in adjacent spatial units), which is calculated as follows:

10 $$Moran's\;I\;=\;\left[\overset n{\underset{i=1}{\sum\;}}\sum_{\;j=1}^n\;w_{ij}\left(Y_i-\overline Y\right)\left(Y_i-\overline Y\right)\right]/\left(S^2\;\overset n{\underset{i=1}{\sum\;}}\sum_{\;j=1}^n\;W_{ij}\right)$$

In the formula, ${S}^{2}=\frac{1}{\mathrm{n}}\sum\nolimits_{i=1}^{n}\left({Y}_{i}-\overline{Y }\right)$, $\overline{Y }=\frac{1}{\mathrm{n}}\sum\nolimits_{i=1}^{n}{Y}_{i}$, ${Y}_{i}$ is the observed value (i.e., carbon emissions per capita) of province *i*; *n* is the number of provinces. ${w}_{ij}$ is the spatial adjacency weight between provinces. Moran’s *I* indicates the global spatial autocorrelation of provincial carbon emissions per capita, and its value range is − 1 ≤ *I* ≤ 1. When *I* is close to − 1, it means that per capita carbon emissions are spatially negatively correlated among provinces; when *I* is close to 1, it means that per capita carbon emissions are spatially positively correlated; and when *I* is equal to 0, it means that there is no spatial autocorrelation.

**Fig. 1:**
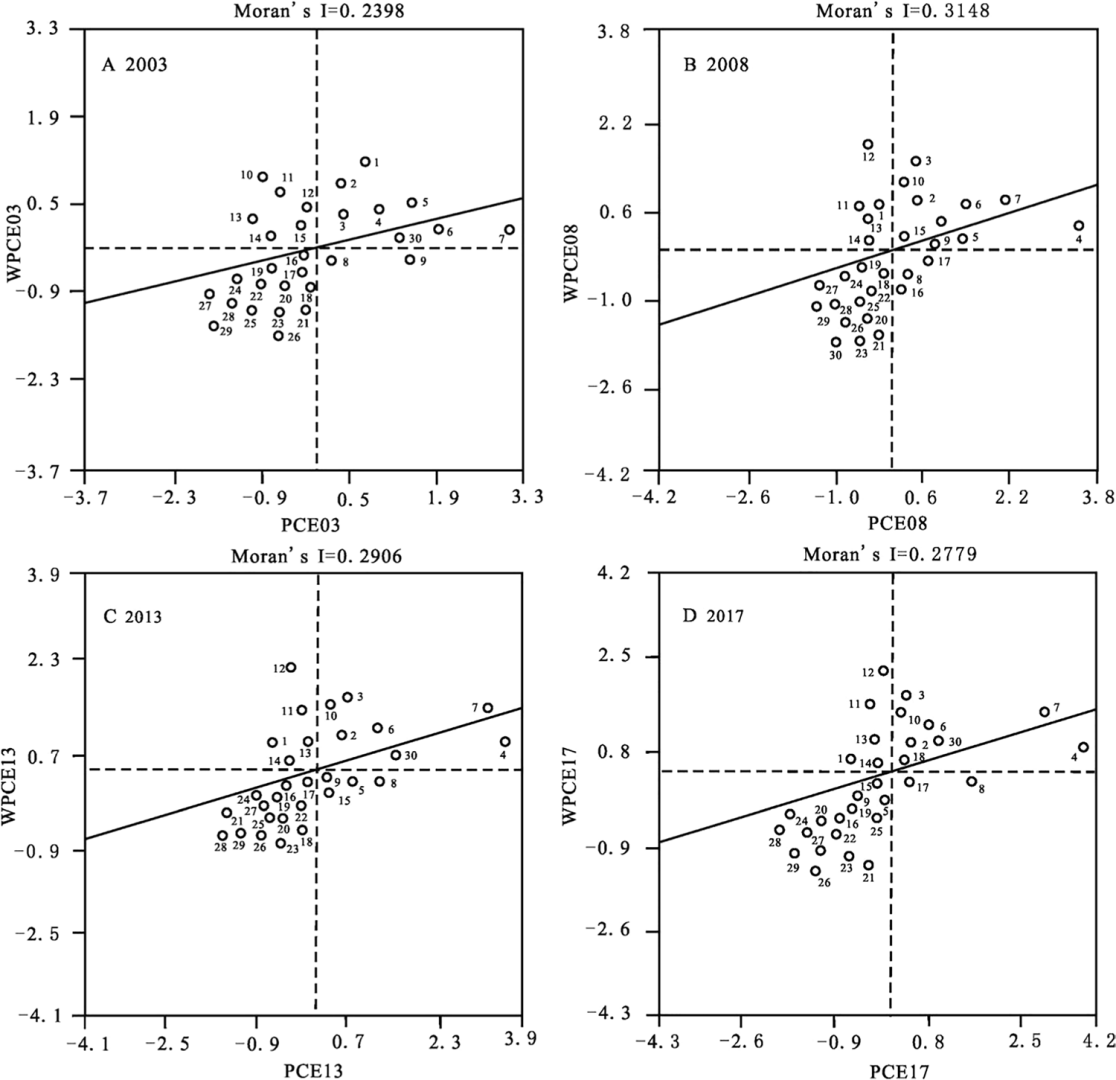
Moran scatter plot of China’s provincial carbon emission per capita in typical years (The Arabic numerals in the figure represent provinces. 1―Beijing; 2―Hebei; 3―Liaoning; 4―Innere Mongolei; 5―Tianjin; 6―Shanxi; 7―Ningxia; 8―Xinjiang; 9―Shangshai; 10―Jilin; 11―Gansu; 12―Heilongjiang; 13―Shaanxi; 14―Henan; 15―Jiangsu; 16―Zhejiang; 17―Shandong; 18―Qinghai; 19―Anhui; 20―Hubei; 21―Guizhou; 22―Fujian; 23―Guangdong; 24―sichuan; 25―Chongqing; 26―Yunnan; 27―Jiangxi; 28―Hunan; 29―Guangxi; 30―Hainan)

From Table 3, it can be seen that the global Moran’s I of China’s provincial carbon emissions per capita has passed the significance test of 5% level, with Z-values above 2.5 and Moran’s I values around 0.3, which indicates that provincial carbon emissions per capita are not completely random, but show a significant positive spatial correlation. The local Moran scatter plot (Fig. 1) of China’s provincial carbon emissions per capita also shows that most provinces fall in quadrants I and III. In 2003, 2008, 2013, and 2017, the proportion of provinces in the quadrants I and III accounted for 70.0%, 73.3%, 70.0%, and 76.7%, respectively. This indicates that carbon emissions of most provinces in China have strong spatial dependence in a local space, and the high-high and low-low agglomerations are obvious; that is, the increase (or decrease) in the per capita carbon emissions of the surrounding provinces will drive an increase (or decrease) in the per capita carbon emissions of the region. The above analysis suggests that it is very necessary to consider the spatial spillover effect of carbon emissions in the panel model when exploring the impact of environmental decentralization on carbon emissions.

**Table 3:** Global Moran's I of China’s provincial carbon emissions per capita from 2003 to 2017

Year	Moran’s *I*	E(*I*)	SD(*I*)	Z(*I*)	*P*
2003	0.2398	− 0.0357	0.1173	2.5023	0.02
2004	0.3680	− 0.0357	0.1280	3.2772	0.01
2005	0.3340	− 0.0357	0.1223	3.1516	0.01
2006	0.3450	− 0.0357	0.1175	3.3837	0.01
2007	0.3359	− 0.0357	0.1114	3.4869	0.01
2008	0.3148	− 0.0357	0.1015	3.5903	0.01
2009	0.2853	− 0.0357	0.0989	3.3854	0.02
2010	0.3186	− 0.0357	0.0969	3.7912	0.01
2011	0.2836	− 0.0357	0.0911	3.6169	0.01
2012	0.2874	− 0.0357	0.0935	3.5556	0.01
2013	0.2906	− 0.0357	0.0985	3.4022	0.01
2014	0.2930	− 0.0357	0.1004	3.3597	0.01
2015	0.2840	− 0.0357	0.1012	3.2446	0.02
2016	0.2786	− 0.0357	0.1020	3.1502	0.03
2017	0.2779	− 0.0357	0.1001	3.1992	0.02

E(*I*) is the expected value, $\mathrm{E}\left(I\right)=-1/\left(\mathrm{n}-1\right)$; SD(*I*) is the standard deviation; Z(*I*) is the standardized statistic, $\mathrm{Z}\left(I\right)=\left[I-E\left(I\right)\right]/\sqrt{var\left(I\right)}$; *P* is the significance level of *I*, which is obtained by 1000 times of Monte Carlo simulation. In this study, if the *P*-value is less than the given significance level ($\alpha =0.05$) and $\left|\mathrm{Z}\right|>1.96$, it means that the provincial carbon emissions per capita have significant spatial correlation; otherwise, the spatial correlation is not significant

### Empirical results

#### The impact of environmental decentralization on carbon emissions at the national level

In order to select an appropriate estimation model to eliminate the endogenous problem of variables (for example, instrumental variables, fixed effect model, and propensity score matching can solve the problem of endogenous variables fixed effect model or random effect model), this paper uses stata16.0 software to conduct Hausman test on static panel model, static spatial panel model, and dynamic spatial panel model. The results show that Hausman test rejects the original hypothesis of random effect of the above model at the significance level of 1%. Therefore, the double-fixed effects model (i.e., double fixation of individual and time effect) is selected to explore the impact of environmental decentralization on carbon emissions based on the maximum likelihood method. The estimation results of each model are presented in Table 4. Among them, the columns (1), (3), and (5) are the regression results based on static panel model, static spatial panel model, and dynamic spatial panel model with ED as the core explanatory variable; the columns (2), (4), and (6) are the estimation results after considering FD based on the above model, respectively.

**Table 4:** Basic regression results of environmental decentralization and provincial carbon emissions in China

Variables	Static panel regression		Static spatial panel regression		Dynamic spatial panel regression	
	(1)	(2)	(3)	(4)	(5)	(6)
*L.lnPCO*_*2*_					0.3651^***^ (0.0284)	0.3660^***^ (0.0283)
*ED*	− 0.1267^**^ (0.0659)		− 0.0922^**^ (0.0386)		− 0.1056^***^ (0.0523)	
*FD*		0.1242^***^ (0.0252)		0.1608^***^ (0.0150)		0.1124^***^ (0.0237)
*lnPGDP*	0.9143^***^ (0.1413)	0.8487^**^ (0.4267)	0.4169^***^ (0.1263)	0.3536^***^ (0.1249)	0.1980^***^ (0.0511)	0.1531^***^ (0.0095)
(*lnPGDP*)^2^	− 0.0543 (0.0354)	− 0.0442 (0.0731)	− 0.0054 (0.0216)	0.0031 (0.0216)	− 0.0126 (0.0188)	− 0.0187 (0.0189)
*lnPD*	− 0.1475^*^ (0.0825)	− 0.1138^*^ (0.06970)	− 0.0192 (0.1209)	− 0.0215 (0.1788)	− 0.1567 (0.1584)	− 0.1295 (0.1562)
*RD*	− 0.0660^***^ (0.0207)	− 0.0663^*^ (0.0413)	− 0.1044^***^ (0.0194)	− 0.1052^***^ (0.0194)	− 0.0616^***^ (0.0173)	− 0.0621^***^ (0.0174)
*FDI*	− 0.0154^**^ (0.0065)	− 0.0125 (0.0110)	− 0.0099^**^ (0.0042)	− 0.0081 (0.0051)	− 0.0051 (0.0045)	− 0.0038 (0.0045)
*lnINDUS*	0.2351^**^ (0.0849)	0.2413^***^ (0.0741)	0.2986^**^ (0.0726)	0.3155^***^ (0.0736)	0.1594^***^ (0.0541)	0.1716^***^ (0.0650)
*OPEN*	0.1154^*^ (0.0771)	0.0810 (0.1312)	0.1126^*^ (0.0697)	0.0793 (0.0665)	0.1182^**^ (0.0604)	0.1364^**^ (0.0576)
W*lnPCO_2_			0.5328^***^ (0.1149)	0.5517^***^ (0.1143)	0.2739^**^ (0.1140)	0.2864^**^ (0.1135)
*Rho value*			0.3325^***^ (0.1012)	0.3264^***^ (0.1043)	0.3491^***^ (0.1274)	0.3376^***^ (0.1263)
*Constants* *R*^2^	− 0.3704^*^ (0.4568) 0.7205	− 0.6521 (0.6601) 0.7153	0.8183	0.8178	0.8620	0.8619
Log–L			315.51	314.88	377.41	377.19
IE/TE	Y/Y	Y/Y	Y/Y	Y/Y	Y/Y	Y/Y
*Obs*	450	450	450	450	450	450

*, **, and *** indicate significance at the levels of 10%, 5%, and 1% levels, respectively. The values in parentheses are standard errors. *W* indicates geographic adjacency weight matrix. IE and TE represent individual effect and time effect respectively. *Y* represents that variables or effects are controlled

Table 4 shows that in columns (1), (3), and (5), the estimated coefficients of the impact of environmental decentralization on provincial carbon emissions are negative and significant at the 5% level indicating that environmental decentralization is beneficial in reducing China’s carbon emissions. This result can be explained from two aspects. First, environmental decentralization has given local government greater autonomy in environmental management. Compared with the centralization, local governments have stronger ability to obtain local information, so they can better understand the environmental needs of local residents and achieve rational resource allocation with lower costs and information advantages, and to formulate targeted environmental policies in terms of emission reduction and green technology, thereby promoting the coordinated development of the local economy and the environment. Secondly, as the degree of environmental decentralization increases, the number of local environmental protection personnel will increase. While promoting the gradual formation of local environmental regulatory networks, local governments can make real-time environmental policy adjustments according to local environmental conditions, thus ultimately improving local environmental quality. However, it is surprising that the results of this study on the impact of environmental decentralization on carbon emissions are inconsistent with the results of Lu and Zhang (2016) and Zhang et al. (2017a, b). In other words, the decentralized environmental management system is not conducive to carbon emission control, on the contrary, will aggravate carbon emissions with the increasing degree of environmental decentralization. They explained this result from the perspective of jurisdictional competition as that environmental decentralization would cause local governments to trigger “race to the bottom,” which resulted in ineffective environmental regulation, thereby increasing carbon emissions. This is consistent with Sigman’s (2014) conclusion that decentralization may lead to the inefficiency of environmental policy. Although their explanation seems to be reasonable, the conclusion is still debatable. First, their research period mainly focused on 1992–2010 (Lu and Zhang 2016; Zhang et al. 2017a, b), while this study spanned the period 2000–2017. With the change of time, the era conditions of the impact of environmental decentralization on carbon emissions are changing. Especially, since 2007, the Chinese government has gradually incorporated energy conservation and emission reduction into the local performance appraisal system. Under the background of lifelong accountability and one-vote veto system for local officials, local governments’ awareness of environmental protection has been strengthened. Local officials can no longer relax environmental regulations to attract foreign investment as they did in the past, which makes the GDP championship that local governments have always emphasized economic growth and ignored environmental protection due to the loss of environmental institutional foundation. Therefore, we can speculate that the conclusions of Lu and Zhang (2016) and Zhang et al. (2017a, b) that environmental decentralization aggravates carbon emissions may be related to their earlier study period. Second, the local carbon emission reduction incentive mechanism is gradually formed under the decentralized environmental management system. With the increasing carbon emissions in recent years, China is under pressure to fulfill its commitment of independent emission reduction in 2030. To this end, the Chinese government attaches great importance to the governance of carbon emissions, and gradually takes the effective curbing of carbon emissions as an important yardstick to evaluate the political performance of local officials, combining this with economic incentives. This has mobilized the initiative of local officials to implement carbon emission reduction to a certain extent. It means that local governments are given more over environmental management power, which may enable them to formulate more accurate environmental regulations and reasonable investment in environmental governance according to the local ecological environment and economic development level, thus forming an incentive and compatibility mechanism to effectively curb carbon emissions and solve the problems of inconsistent goals and information asymmetry between the central and local governments in carbon emission management. Therefore, in the case of carbon emissions, a modest decentralization will be conducive to carbon emissions governance, while over-centralization may result in increased carbon emissions.

In terms of the effect of fiscal decentralization on carbon emissions, the estimated coefficients of fiscal decentralization are significantly positive at the 1% level in columns (2), (4), and (6), which is consistent with the previous results that the improvement of fiscal decentralization will significantly increase carbon emissions (He 2015; Zhang et al. 2011). This may be due to fiscal decentralization significantly reducing local governments’ efforts and investment in environmental governance, thus hindering the innovative development of low-carbon environmental technologies. Although increased financial autonomy helps to stimulate the enthusiasm of local governments to develop regional economy to a certain extent, this official promotion model based on GDP growth is usually at the expense of the environment (Yan 2012; Wang and Zhang 2014). The abovementioned positive correlation between fiscal decentralization and carbon emissions shows that financial autonomy is not suitable for local government as much as environmental decentralization. As previously analyzed, if the fiscal decentralization is used as a simple indicator to measure the environmental decentralization, it will be difficult to truly reflect the impact of environmental decentralization on carbon emissions. Therefore, the direct exploration of the relationship between environmental decentralization and carbon emissions from the internal logic of environmental management is more suitable for evaluating the effect of Chinese-style environmental federalism.

Further, from the regression results of the static and dynamic spatial panel models (Table 4), the estimated coefficients of the spatial lag (W*lnPCO_2_) are both significantly positive at least at the 5% level, indicating that China’s provincial carbon emission has significant path dependence. That is, the carbon emission of any province will be influenced by the carbon emissions of the neighboring areas. If the spatial correlation is ignored, there will be deviation in the estimation results. In addition, the first-order lagged term of the explained variable is positively correlated with the carbon emissions of the current period at the 1% level, which indicates that the per capita carbon emissions in each province have obvious continuity and stickiness in time, thus highlighting that carbon emissions have a certain inertia-dependent feature (Zhang et al. 2017a, b). That is to say, the carbon emissions remaining in the atmosphere in the previous period may aggravate the carbon emissions of the region in the current period. Therefore, if short-term carbon emissions are not treated timely and effectively, it will lead to long-term and more costly negative environmental effects.

As for the control variables, this study mainly interprets the regression results of column (5) in the dynamic spatial panel. Table 4 reveals that the estimated coefficients of the economic growth are significantly positive; indicating that in China’s economic transition period, with rapid economic development, the increase of energy consumption will significantly increase provincial carbon emissions. It is noteworthy that the squared term coefficient of economic growth is negative but not significant, which indicates that there is an inverted U-shaped Kuznets curve between economic growth and provincial carbon emissions. This means that when the economic development reaches a certain level, residents’ demand for environmental quality will become higher and higher, and local governments will provide some financial and policy support to effectively control carbon emissions, so as to improve environmental quality, but the current effect is not obvious. The relationship between population density and carbon emissions is insignificant but negative, indicating that an increase in population density decreases provincial carbon emissions. The reason for this is that the carbon emissions in this study were measured on a per capita basis, which is not inconsistent with previous findings that population growth contributes to increased carbon emissions (Zhu et al. 2010). Of course, regions with higher population densities usually have more technical and talent capital, which is conducive to the rapid development of energy-saving and emission reduction technologies, so as to reduce carbon emissions. The estimated coefficient of R&D intensity is significantly negative at the 1% level, indicating that R&D intensity has a significant inhibitory effect on carbon emissions, which is consistent with the findings of Cole et al. (2013) and Han (2018), implying that improving energy efficiency by inducing low-carbon technological progress through science and technology innovation is an important means to curb carbon emissions growth and effectively promote the achievement of carbon emission reduction targets in China. The estimated coefficient for FDI is negative and fails to pass the significance test, indicating that the inhibitory effect of FDI on carbon emissions is not obvious. This may be due to the fact that China is currently undergoing a transition from quantity to quality in the draught of foreign capital. Under the influence of “pollution refuge” and “pollution halo” effects, the carbon emission reduction effect brought by FDI due to technology transfer may be partially offset by its negative environmental effect, resulting in the insignificant impact of FDI on curbing carbon emissions. The impact of industrial structure on carbon emissions, as measured by the proportion of industry, is significantly positive at the 1% level, indicating that the high proportion of secondary industries is an important factor aggravating carbon emissions. This also means that although China is currently accelerating the transformation, upgrading and greening of industrial structure, it has not fundamentally reversed the extensive economic growth model of industrial development. The growth of industrial output is still at the expense of massive primary energy consumption and environmental sacrifice (Zhang and Wang 2020), which leads to increased carbon emissions. Therefore, the development of new and strategic industries based on clean production and the reduction of dependence on resource-based industries are still important means to reduce carbon emissions. In addition, the effect of trade openness on provincial carbon emissions is also significantly positive, which indicates that trade openness is not conducive to energy conservation and emission reduction. The reason may be related to the negative function of trade openness in transferring environmental pollution. It has been reported that environmental pollution will be transferred through trade openness from areas with stronger environmental regulations to areas with weaker environmental regulations, in which looser environmental regulation will usually promote economic growth and bring more carbon emissions (Poon et al. 2006).

#### The impact of environmental decentralization decomposition index on China’s carbon emissions

In order to further explore the impact of environmental decentralization on provincial carbon emissions, the three decomposition indicators of environmental decentralization are re-estimated by dynamic spatial model. The results in Table 5 show that the estimated coefficient of environmental administrative decentralization is negative and significant at the 1% level, indicating that environmental administrative decentralization is beneficial to local environmental administrations to reasonably arrange environmental governance investment and timely adjust environmental policies according to the local socio-economic and ecological conditions, thus forming a “race to the top” in environmental administrative matters and finally reduce local carbon emissions. The estimated coefficient of environmental monitoring decentralization is also significantly negative at the 1% level, indicating that the decentralization of environmental monitoring will also suppress local carbon emissions. This is because local governments have more advantages than the central government in environmental quality monitoring, assessment, and early warning. Therefore, a moderate increase in environmental monitoring decentralization is more conducive to local environmental protection departments to effectively carry out environmental monitoring activities and environmental quality assessment, thus providing more accurate environmental quality information for environmental administration and environmental supervision departments to some extent, and thereby improving environmental quality and reducing carbon emissions. It is noteworthy that the absolute value of the estimated coefficient of the environmental monitoring decentralization is the smallest among the three decomposers, which may be related to the indirect effect of environmental monitoring on carbon emission mainly by providing information for environmental administration and environmental monitoring. In contrast to the above two, the estimated coefficient for the environmental supervision decentralization is significantly positive at the 1% level, indicating that the environmental supervision decentralization is not conducive to carbon emission reduction. This is because environmental supervision department is the most direct pollution emission control organization, and its environmental supervision services such as environmental enforcement and environmental supervision may face great resistance when implemented locally. When there is a conflict between environmental supervision and local economic interests, local environmental protection departments will be interfered by local governments in the pursuit of economic growth, and relax environmental regulations in terms of emissions declaration, environmental project acceptance, and environmental enforcement and inspection, thus increasing carbon emissions. Therefore, on the premise of advocating green GDP and reforming performance assessment standards, the power of environmental supervision should be appropriately shifted upward, and supplemented by the coordination and supervision of the central government. Only in this way can we give play to the inhibitory effect of environmental supervision on carbon emissions and avoid the “race to the bottom” of carbon emissions by local governments for economic development.

**Table 5:** The regression results of different environmental decentralization and provincial carbon emissions

Variables	Dynamic spatial panel regression		
	(1)	(2)	(3)
*EAD*	− 0.0814^***^(0.0108)		
*ESD*		0.0725^***^(0.0228)	
*EMD*			− 0.0306^***^(0.0086)
Control Variables	Y	Y	Y
*L.lnPCO*_2_	0.3540^***^(0.0283)	0.3509^***^(0.0281)	0.3547^***^(0.0284)
W*lnPCO_2_ *Rho* value *R*^2^	0.3402^***^(0.1123) 0.3563^***^ (0.1264) 0.8646	0.3498^***^(0.1121) 0.3547^***^ (0.1263) 0.8674	0.3415^***^(0.1123) 0.3577^***^ (0.1264) 0.8645
*Log-L*	381.74	386.43	381.48
IE/TE	Y/Y	Y/Y	Y/Y
*Obs*	450	450	450

*, **, and *** indicate significance at the levels of 10%, 5%, and 1% levels, respectively. The values in parentheses are standard errors. *W* indicates geographic adjacency weight matrix. IE and TE represent individual effect and time effect respectively. *Y* represents that variables or effects are controlled

#### The impact of interaction of environmental decentralization and fiscal decentralization on China’s carbon emissions

After adding the interaction term of environmental decentralization and fiscal decentralization variables into the dynamic spatial panel model, the results obtained by regression with carbon emissions are listed in Table 6. Table 6 shows that the regression coefficients of the interaction terms of environmental decentralization and fiscal decentralization, environmental administrative decentralization and fiscal decentralization, environmental monitoring decentralization and fiscal decentralization, and environmental supervision decentralization and fiscal decentralization are all significantly positive at the 5% level. It shows that the combination of various environmental decentralization and fiscal decentralization can promote carbon emissions, which means that the inhibitory effect of environmental decentralization on carbon emissions will be constrained by fiscal decentralization to some extent. The reason for this is that fiscal decentralization gives local governments more financial autonomy and economic incentives, while environmental decentralization gives local governments the power to protect and manage the environment. When local governments have the financial autonomy delegated by the central government and the ability to intervene local environmental matters, they usually sacrifice the environment for rapid economic growth, and even take the way of “free riding” and collusion between government and enterprises. When the environmental degradation effect of fiscal decentralization is greater than the inhibitory effect of environmental decentralization on carbon emissions, carbon emissions will be intensified. The empirical results in this study support the findings of Tian and Wang (Tian and Wang 2018). Although the relationship between fiscal decentralization and carbon emissions has been discussed in many studies (Zhang et al. 2011; Ran et al. 2020; Yan 2012; Wang and Zhang 2014), this study is more relevant because it is in line with the theory of environmental federalism.

**Table 6:** The impacts of the interaction between environmental decentralization and fiscal decentralization on China’s provincial carbon emissions

Variables	*X* = *ED*	*X* = *EAD*	*X* = *ESD*	*X* = *EMD*
*L.lnPCO*_*2*_	0.3512^***^(0.0285)	0.3543^***^(0.02836)	0.3485^***^(0.0279)	0.3558^***^(0.0284)
*X*	− 0.1007^**^(0.0507)	− 0.1635^**^(0.0680)	0.0731^**^(0.0267)	− 0.1491^**^(0.0542)
*FD*	0.1301^***^(0.0171)	0.0933^***^(0.0211)	0.1163^***^(0.0241)	0.1162^***^(0.0323)
X*FD	0.0784^**^(0.0146)	0.0643^**^(0.0184)	0.0587^**^(0.0180)	0.0574^**^(0.0201)
Control Variables	Y	Y	Y	Y
W*lnPCO_*2*_	0.3283^***^(0.1127)	0.3379^***^(0.1124)	0.3440^***^(0.1121)	0.3467^***^(0.1125)
*Rho* value	0.3487^***^(0.1267)	0.3558^***^ (0.1258)	0.3549^***^ (0.1261)	0.3564^***^ (0.1264)
*R*^2^	0.8652	0.8650	0.8683	0.8560
Log-L	382.63	382.33	387.98	382.34
IE/TE	Y/Y	Y/Y	Y/Y	Y/Y
*Obs*	450	450	450	450

*, **, and *** indicate significance at the levels of 10%, 5%, and 1% levels, respectively. The values in parentheses are standard errors. *W* indicates geographic adjacency weight matrix. IE and TE represent individual effect and time effect respectively. *Y* represents that variables or effects are controlled. ED, EAD, ESD and EMD represent overall environmental decentralization, environmental administration decentralization, environmental supervision decentralization, and environmental monitoring decentralization, respectively

In addition, comparing the regression coefficients of the interaction terms in Table 6, it is found that the coefficient of the overall environmental decentralization is the largest, followed by environmental administration decentralization, and the coefficients of environmental supervision decentralization and environmental monitoring decentralization are the smallest. The reason for this difference may be related to the allocation of environmental powers set by the environmental authorities. The environmental system department, which is the prime minister’s agency for environmental governance, has the highest estimated interaction coefficients because its environmental powers are most affected by the increase in financial autonomy; the environmental administration department, which is responsible for formulating environmental policies and coordinating resource allocation, has the second-highest estimated interaction coefficients because these powers are weakened to a large extent by financial decentralization, which in turn affects the inhibitory effect of environmental decentralization on the growth of carbon emissions; and the environmental monitoring department and the environmental supervision department, which are the concrete implementers of environmental protection, are less affected by fiscal decentralization because their functions are non-substitutional, so their interaction coefficients are smaller.

#### The impact of environmental decentralization on carbon emissions in different regions of China

Considering the spatial heterogeneity of environmental decentralization in different regions and the differences in resource endowment, economic development, and technological innovation, we explored the heterogeneity of environmental decentralization’s impact on carbon emissions in eastern, central, and western regions of China. Table 7 shows that except for environmental supervision decentralization, the regression coefficients of overall environmental decentralization and its decomposition indicators in the eastern region are negative but not significant, indicating that the decentralization of environmental management in this region is conducive to reducing carbon emissions, but the emission reduction effect is not fully apparent. The reason may be that the economy in the eastern region is relatively developed, and technological innovation and human capital have a strong driving effect on industrial transformation. In addition, the local governments in the area have a strong awareness of environmental protection, and the distribution of grass-roots environmental protection personnel has a high level, which weakens the inhibitory effect of environmental decentralization on carbon emissions. The estimated coefficients on the environmental decentralization, environmental administrative decentralization, and environmental supervision decentralization are significantly positive at the 10% level in the central region, suggesting that environmental decentralization in the central region contributes to the increase in carbon emissions. Although environmental monitoring decentralization helps to curb carbon emissions in this region, overall decentralization of environmental management matters are not conducive to carbon reduction. This may be related to the relative lag of economic development in the central region and the greater impact of economic incentives on local governments. And environmental decentralization facilitates local governments to focus more on economic development and at the expense of the environment. Therefore, in the central region, environmental management powers (such as environmental administration power and environmental supervision power) should be appropriately transferred upwards to avoid the adverse impact of excessive environmental decentralization on carbon emission reduction. The coefficients of overall environmental decentralization, environmental administrative decentralization, and environmental monitoring decentralization in western China are all significantly negative at the 5% level, while environmental supervision decentralization has a significant positive correlation with the growth of carbon emissions (Table 7), indicating that environmental decentralization has a greater impact on carbon emissions in western region than in eastern and central regions. The reason is that the western region is currently in the transition from the stage of laying the foundation for western development (2000–2010) to the stage of accelerated development (2010–2030), and the contradiction between economic development and resources and environment is the most intense. In recent years, in addition to adjusting the industrial structure, improving the investment environment and developing science, technology, and education, the central government has increased its intervention in the ecological and environmental protection of western regions, and taken the environmental protection of key ecological function areas in western regions as an important indicator for local performance appraisals. At the same time, local governments in the western region are given sufficient incentives for environmental protection and necessary policy support. In this context, once the environmental management is decentralized, it will make up for the shortcomings of the past grass-roots environmental management system, which will promote the gradual formation of a relatively perfect environmental supervision mechanism. Consequently, local governments can formulate environmental policies and control carbon emissions on time with the gradually emerging advantages of information and resource allocation. As a result, the increase in environmental decentralization in the west has a greater dampening effect on carbon emissions than in the east and central regions. It is worth mentioning that the regression coefficients of environmental supervision decentralization in the eastern, central, and western regions are positive, but only in the western region passed the significance test of 5%, indicating that the promotion effect of environmental supervision decentralization on the growth of carbon emissions is more pronounced in the western region. The reason is that the economic development level of the western region is lower than that of the eastern and central regions, and the local governments are more stimulated by economic growth. The contradiction between environmental supervision and local economic development is greater than that in the eastern and central regions, resulting in greater resistance to the implementation of environmental supervision in the western region and ultimately affecting the supervision effect. Therefore, it is necessary to appropriately transfer the power of environmental supervision upwards and vertically manage environmental inspection matters.

**Table 7:** dynamic spatial regression results of environmental decentralization and its interaction with administrative decentralization on carbon emissions in different regions of China

Variables	Eastern region				Central region				Western region			
	*X* = *ED*	*X* = *EAD*	*X* = *ESD*	*X* = *EMD*	*X* = *ED*	*X* = *EAD*	*X* = *ESD*	*X* = *EMD*	*X* = *ED*	*X* = *EAD*	*X* = *ESD*	*X* = *EMD*
*L.lnPCO*_2_	0.0816^**^ (0.0468)	0.1402^***^ (0.0460)	0.0993^**^ (0.0465)	0.0928^**^ (0.0434)	0.2048^***^ (0.0785)	0.2142^***^ (0.0781)	0.1929^***^ (0.0781)	0.2181^***^ (0.0785)	0.2537^***^ (0.0477)	0.2593^***^ (0.0490)	0.2581^***^ (0.0491)	0.2570^***^ (0.0483)
*X*	− 0.1399 (0.1483)	− 0.1024 (0.0980)	0.1016 (0.1077)	− 0.0714 (0.1442)	0.2545^*^ (0.1094)	0.4120^*^ (0.2103)	0.2584^*^ (0.1612)	− 0.0762^*^ (0.0384)	− 0.3023^**^ (0.1020)	− 0.1355^**^ (0.0461)	0.1346^**^ (0.0385)	− 0.0933^**^ (0.0273)
*FD*	0.0424 (0.0864)	0.02315 (0.0384)	0.0142 (0.0412)	0.0356 (0.0885)	0.1581^**^ (0.0419)	0.2105^***^ (0.0228)	0.1490^**^ (0.0208)	0.1329^**^ (0.0126)	0.0527^*^ (0.0369)	0.0312^*^ (0.0215)	0.0221^*^ (0.0146)	0.0394^*^ (0.0285)
X*FD	− 0.0698 (0.1010)	− 0.1025 (0.0501)	− 0.0470 (0.0651)	− 0.0481 (0.0890)	− 0.0572 (0.1122)	− 0.1618 (0.1104)	0.0684 (0.0763)	0.0446 (0.0522)	0.0387^**^ (0.0113)	0.0289^**^ (0.0078)	0.0741^**^ (0.0391)	0.0257^**^ (0.0171)
Control Variables	Y	Y	Y	Y	Y	Y	Y	Y	Y	Y	Y	Y
W*lnPCO_2_ *Rho* value *R*^2^	0.1327 (0.1398) 0.2625 (0.1769) 0.9017	0.1686 (0.1411) 0.2632^*^ (0.1767) 0.8959	0.1205 (0.1413) 0.2637^*^ (0.1766) 0.8983	0.1887 (0.1393) 0.2631^*^ (0.1766) 0.9057	0.2240^**^ (0.1038) 0.2926^*^ (0.1674) 0.8556	0.2765^**^ (0.1064) 0.3028^*^ (0.1675) 0.8578	0.2042^**^ (0.1027) 0.2952^*^ (0.1677) 0.8575	0.2485^**^ (0.0516) 0.2943^*^ (0.1676) 0.8556	0.5071^***^ (0.1778) 0.4527^***^ (0.1769) 0.9082	0.5280^***^ (0.1796) 0.4536^***^ (0.1771) 0.9025	0.5352^***^ (0.1785) 0.4548^***^ (0.1776) 0.9041	0.5279^***^ (0.1783) 0.4536^***^ (0.1774) 0.9016
Log-L	202.78	198.03	199.99	206.17	102.58	103.52	103.38	102.61	143.60	136.54	136.51	137.91
IE/TE	Y/Y	Y/Y	Y/Y	Y/Y	Y/Y	Y/Y	Y/Y	Y/Y	Y/Y	Y/Y	Y/Y	Y/Y
*Obs*	165	165	165	165	120	120	120	120	165	165	165	165

The eastern region includes 11 provinces (or municipalities directly under the central government) of Beijing, Tianjin, Hebei, Liaoning, Shanghai, Jiangsu, Zhejiang, Fujian, Shandong, Guangdong, and Hainan. The central region includes 8 provinces of Shanxi, Jilin, Heilongjiang, Anhui, Jiangxi, Henan, Hubei, and Hunan. The western region includes 11 provinces (or autonomous regions) of Inner Mongolia, Guangxi, Chongqing, Sichuan, Guizhou, Yunnan, Shaanxi, Gansu, Qinghai, Ningxia, and Xinjiang. *, **, and *** indicate significance at the levels of 10%, 5%, and 1% levels, respectively. The values in parentheses are standard errors. *W* indicates geographic adjacency weight matrix. *Y* represents that variables or effects are controlled

Table 7 shows that the impacts of fiscal decentralization on carbon emissions are positive in the eastern, central and western regions, indicating that fiscal decentralization contributes to the growth of carbon emissions, but the extent to which fiscal decentralization affects carbon emissions varies across regions. In the eastern region, there is no significant positive correlation between fiscal decentralization and carbon emissions, and the regression coefficients are the smallest, while in the central and western regions, the two show significant positive correlation at the level of at least 5% and 10%, respectively. The reasons for this difference may be related to the different incentives formed by regional economic development and financial level, as well as the differences in resource endowments due to the ecological situation. In the eastern region, economic development and financial resources are higher, and local governments prefer the environment over economic growth incentives. Therefore, in the context of fiscal decentralization, local environmental protection departments resolutely implement environmental protection policies, so that fiscal decentralization has little influence on environmental management matters, thus resulting in weak effects on carbon emissions growth. In the central region, due to the relative lag in economic development, the incentive given to local governments to develop economy is far greater than the preference of local governments for environment. Thus, as fiscal autonomy increases, local governments make way for economic development by reducing environmental regulations or distorting environmental policies, resulting in the strongest contribution of fiscal decentralization to increasing carbon emissions. As for the western region, although the level of economic development is the lowest and local governments are also influenced by the incentives given by fiscal decentralization to develop economy, the central government have paid more attention to environmental issues and given sufficient incentives for environmental protection due to the fragile ecological environment. Therefore, even though local governments still have strong incentives for economic development in the western region, the behavior of sacrificing the environment for economic development has been curbed to a great extent. As a result, the regression coefficient of the impact of fiscal decentralization on carbon emissions is small and only significantly positive at the 10% level.

The impacts of the interaction term between environmental decentralization and its decomposition variables and fiscal decentralization on regional carbon emissions show regional heterogeneity (Table 7). In the eastern region, the coefficients of the interaction terms are all negative, indicating that the combination of environmental decentralization and its disaggregated variables with fiscal decentralization have a restraining effect on carbon emissions, while in the central and western regions, the coefficients of the interaction terms are all positive and significant in the western region, except for the central region, where the coefficients of the interaction terms of environmental decentralization and environmental administrative decentralization with fiscal decentralization are negative, indicating that the interaction terms between most environmental decentralization variables and fiscal decentralization in the central and western regions promote carbon emissions. This is mainly because local governments in the central and western regions have stronger incentives to pursue economic growth than those in the eastern regions, and even squeeze out environmental protection spending for high-return productive investments, thus leading to the increased obstruction of fiscal decentralization to local environmental management. In the economically developed eastern regions, local governments prefer the environment and have sufficient funds to control environmental pollution. Hence, fiscal decentralization interferes less with environmental management matters, resulting in the inhibitory effect of the interaction terms on carbon emissions.

In addition, the period lag coefficients, spatial correlation coefficients, and the effects of other control variables on carbon emissions are all consistent with the results in Table 4, which will not be repeated here. It is worth noting that the spatial correlation coefficients of carbon emissions in the eastern region are positive but not significant, while the ones in the central and western regions are significantly positive at the level of 5% (Table 7). This reflects that there is still an obvious path dependence of carbon emissions in the central and western regions, while the positive spatial correlation of carbon emissions in the eastern region is weak. The reason may be that the high-tech and efficient resources in the eastern region continue to flow to Beijing, Tianjin, Shanghai, and other regions, in which the industries with high energy consumption and high emission are gradually transferred to provinces with low environmental regulation, resulting in a significant decline in carbon emissions, while the carbon emissions in relatively backward eastern provinces such as Liaoning, Hebei, and Fujian decline less (Liu et al. 2018), thus weakening the positive spatial correlation of carbon emissions within the east region. The economic development of the central and western regions mainly relies on the region’s resource endowments and the transfer of industries from the eastern region, with obvious synergistic development effects, resulting in a strong spatial dependence of carbon emissions.

### Robustness test of the impact of environmental decentralization on carbon emissions

In order to test the robustness of the above empirical results, this study re-measures the environmental decentralization and its three decomposition indexes without considering the economic scale reduction factor, and regress them with provincial carbon emissions. The results are listed in Table 8. Compared with the results in Tables 4, 5, and 6, it is found that the relationships between ED, EAD, EMD, and ESD and carbon emissions without considering the economic scale reduction factor all remain stable. Although the regression coefficients of other variables change in varying degrees, the change direction and significance are basically consistent with the above results, which indicates that the estimation results have good robustness.

**Table 8:** The results of a robustness test

Variables	*X* = *ED*	*X* = *EAD*	*X* = *ESD*	*X* = *EMD*
*L.lnPCO*_2_	0.3523^***^ (0.0284)	0.3538^***^ (0.0284)	0.3469^***^ (0.0283)	0.3540^***^ (0.0284)
*X*	− 0.1169^***^ (0.0128)	− 0.0110^***^ (0.0036)	0.0723^***^ (0.0268)	− 0.0531^***^ (0.0126)
*FD*	0.1016^***^ (0.0128)	0.0812^***^ (0.0129)	0.0918^***^ (0.0103)	0.1134^***^ (0.0129)
Control variables	Y	Y	Y	Y
W*lnPCO_2_ *Rho* value	0.3278^***^ (0.1134) 0.3567^***^ (0.1265)	0.3386^***^ (0.1129) 0.3560^***^ (0.1262)	0.3519^***^ (0.1127) 0.3550^**^ (0.1263)	0.3398^***^ (0.1129) 0.3572^***^ (0.1264)
*R*^2^	0.8647	0.8644	0.8666	0.8644
Log-L	381.90	381.43	384.98	381.38
IE/TE	Y/Y	Y/Y	Y/Y	Y/Y
*Obs*	450	450	450	450

*, **, and *** indicate significance at the levels of 10%, 5%, and 1%, levels, respectively. The values in parentheses are standard errors. *W* indicates geographic adjacency weight matrix. IE and TE represent individual effect and time effect respectively. *Y* represents that variables or effects are controlled

## Conclusions and policy recommendations

To achieve the carbon emission reduction target set in 2030, it is necessary to construct a reasonable environmental management system for carbon emissions among government levels. This article empirically examined environmental decentralization and its impact on carbon emissions in the context of fiscal decentralization by constructing a dynamic spatial panel model using inter-provincial panel data from 2003 to 2017 in China. The results show that (1) the period lag and spatial lag coefficients of carbon emissions are both significantly positive, indicating that there is an obvious inertia dependence and spatial path dependence of carbon emissions in China, with high-high and low-low aggregation characteristics. (2) At the national level, considering the spatial spillover effect of carbon emissions, the overall environmental decentralization, environmental administrative decentralization, and environmental monitoring decentralization have a significant and stable negative impact on carbon emissions, indicating that environmental administrative decentralization, environmental monitoring decentralization, and overall environmental decentralization are conducive to reducing carbon emissions in China, while environmental supervision decentralization plays a significant and stable role in promoting carbon emissions, implying that, compared with environmental centralization, the current environmental decentralization system is generally conducive to carbon emission control, but environmental supervision decentralization has certain negative effects on carbon emission reduction. Fiscal decentralization significantly exacerbates carbon emissions, because fiscal decentralization is prone to distort incentives and significantly reduces local governments’ efforts to regulate the environment, thus failing to impose effective constraints on carbon emissions; the interaction term coefficients of environmental decentralization and its disaggregated indicators and fiscal decentralization are both significantly positive at the 5% level, showing that the combination of environmental management rights and fiscal autonomy will have a facilitating effect on carbon emissions, implying that fiscal decentralization weakens the incentives of environmental decentralization for environmental protection and thus exacerbates carbon emissions. (3) At the regional level, there is great spatial heterogeneity in the effects of environmental decentralization on carbon emissions in different regions. The suppression effect of environmental decentralization, environmental administrative decentralization, and environmental monitoring decentralization on carbon emissions in the western region is significantly larger than that in the eastern region; similarly, the promotion effect of environmental supervision decentralization on carbon emissions is also more significant than that in the eastern region. In the central region, in addition to the environmental monitoring decentralization which inhibits carbon emissions, environmental decentralization, environmental administration decentralization, and environmental supervision decentralization promote carbon emissions, indicating that the decentralization of environmental management in the central region does not form an effective incentive for carbon emission management in general, and is not conducive to the implementation of carbon emission reduction. The promotion effect of fiscal decentralization in the eastern part of the country is significantly weaker than that in the central and western part of the country, but the combination of environmental decentralization and its decomposition index with fiscal decentralization is significantly better than that in the central and western part of the country in terms of its inhibiting effect on carbon emissions.

Based on the empirical results, four suggestions for China’s carbon emission management were put forward. (1) At the national level, we can appropriately improve the degree of environmental decentralization and further optimize the allocation of environmental managers among governments at different levels, so as to improve the efficiency of local government’s control over carbon emissions. Meanwhile the assessment system of green GDP should be strengthened to prevent the aggravation of carbon emissions due to the excessive combination of fiscal and environmental decentralization. (2) For different types of environmental decentralization, a differentiated degree of decentralization should be adopted. That is, the environmental administrative power and environmental monitoring powers can be appropriately transferred downward in order to make full use of the cost and information advantages of local governments to achieve effective resource allocation in carbon emission management. The power of environmental supervision should be properly centralized to ensure the authority of environmental supervision. (3) Differentiated environmental decentralization strategies should be scientifically formulated in the east, central, and west regions. Specifically, as the eastern regions have obvious advantages in economy, technology, talent, and information, the central government should further delegate environmental administrative power and environmental monitoring power, and improve local environmental protection information disclosure mechanism to ensure the openness and transparency of environmental monitoring data. In the central region, the central government should increase its intervention in local environmental management and appropriately reduce the local government’s environmental monitoring power and the discretion space in environmental policy-making, while a moderate downward transfer of environmental monitoring power can be considered. In the western region, the central government should grant special treatment in terms of environmental decentralization, that is, appropriately increase the environmental administrative power and the number of environmental protection personnel of local government, gradually improve the construction of grass-roots environmental facilities and environmental monitoring capacity, and guide local governments to “compete upward” in carbon emission control. At the same time, we should strengthen the incentives and constraints of local governments on carbon emission reduction. (4) Considering the spatial spillover effects of carbon emissions, the establishment of a cross-regional and cross-sectoral “joint prevention and control” carbon emissions governance mechanism is an important option to avoid local governments “going it alone” and “free-riding” behavior of carbon emissions.

It is worth noting that, due to the limitations of data and environmental decentralization measurement methods, we only explored the carbon emission effects of environmental decentralization from the inter-provincial panel data, while the impact of environmental decentralization on carbon emission at the municipal level is still unknown. Therefore, future research should further improve the indicators for measuring environmental decentralization, explore the impact of environmental decentralization on carbon emissions from the municipal level, and focus on the spatial dependence of environmental decentralization and its nonlinear relationship with carbon emissions in the empirical model, which can make up for the shortcomings of existing research.

## Data Availability

The datasets used and/or analyzed in this study are available from the corresponding author on reasonable request.
